# Three Different Morphologies of Inferior Vena Cava Thrombosis: Case Reports

**DOI:** 10.1155/2014/349213

**Published:** 2014-03-04

**Authors:** Satoshi Okayama, Yasuki Nakada, Shiro Uemura, Yoshihiko Saito

**Affiliations:** First Department of Internal Medicine, Nara Medical University, 840, Shijo-cho, Kashihara, Nara 634-8522, Japan

## Abstract

Inferior vena cava (IVC) thrombosis is a rare but significant complication in hospitalized patients. However, relevant information regarding IVC thrombosis, especially on its morphology, remains scarce. We present three cases of IVC thrombosis, with each showing a different morphology: mural, floating, and small polyp-like thrombus.

## 1. Introduction

Deep venous thromboses (DVTs) and pulmonary embolisms (PEs), commonly described together as venous thromboembolisms (VTEs), are important complications in hospitalized patients. DVTs commonly occur in the deep veins of the lower leg or the proximal veins of the iliofemoral segment [1] and rarely occur in the inferior vena cava (IVC) [2–5]. Some case reports and reviews of IVC thrombosis currently exist; however, little information has been published regarding its morphology. We present three cases of IVC thrombosis, with each showing a different morphology.

## 2. Case Presentations

### 2.1. Case 1

**Figure 1:**
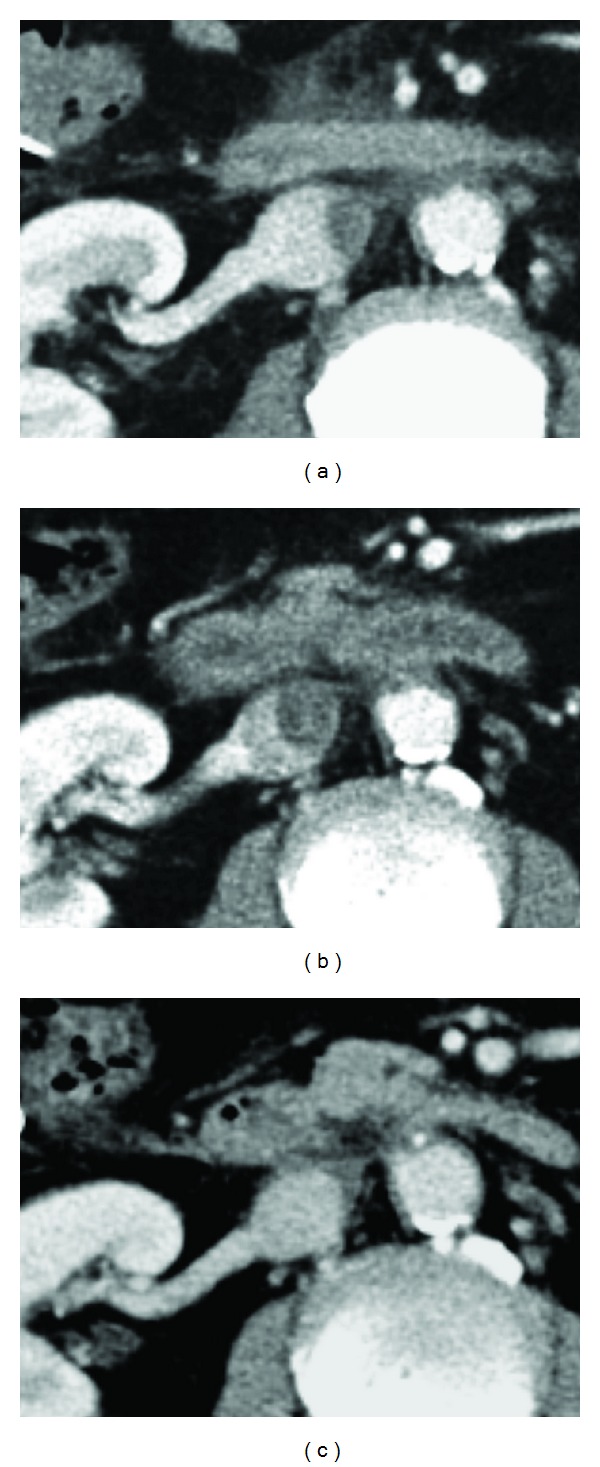
Contrast-enhanced abdominal computed tomography. (a) Mural thrombus extensively adhered to the vessel wall in the infrarenal inferior vena cava (IVC). (b) Anticoagulant therapy with unfractionated heparin and antithrombin was administered, but the IVC thrombus continued to increase in size, five days later. (c) After switching to warfarin and fondaparinux sodium anticoagulant therapy, the IVC thrombosis significantly shrank within one month.

A 60-year-old man underwent surgical resection for cholangiocarcinoma and was referred to our department because of an IVC thrombus detected on contrast-enhanced abdominal computed tomography (CT), four days later. The patient did not have a family history of VTEs or sudden and/or premature deaths. Prior to his diagnosis, he had not complained of chest pain, dyspnea, or lower back pain. His physical examination showed a regular pulse rate of 96 beats/min, a blood pressure of 106/69 mmHg, and a low-grade temperature of 37.8°C. Auscultation failed to detect any obvious murmur or rales, and peripheral leg edema was not observed. Laboratory tests revealed an increased D-dimer concentration (12.8 *μ*g/mL) and a slightly decreased concentration of antithrombin (55%) and protein C (62%). The patient was also negative for the presence of antinuclear antibody, lupus anticoagulant, and cardiolipin antibody; his protein S concentration was normal. Contrast-enhanced abdominal CT demonstrated the presence of a mural thrombus adhered to the vessel wall in the infrarenal IVC (Figure 1(a)); interruption or hypoplasia of the IVC was not observed. We initially administered anticoagulant therapy with unfractionated heparin and antithrombin to minimize the possibility of bleeding complications. However, the IVC thrombus was found to increase in size, five days later (Figure 1(b)). Thus, we switched to an anticoagulant therapy involving warfarin and fondaparinux sodium, and the IVC thrombosis shrank significantly during the following month of treatment (Figure 1(c)); the patient was then discharged.

### 2.2. Case 2

**Figure 2:**
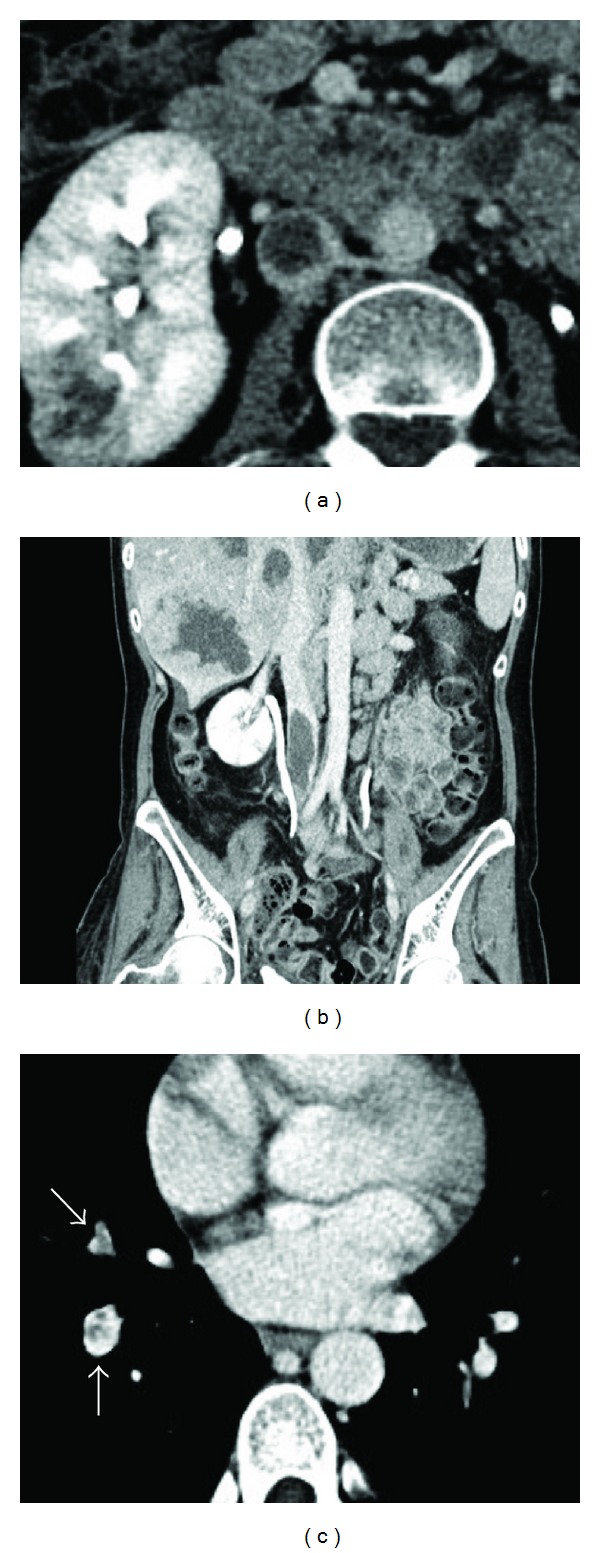
Contrast-enhanced abdominal ((a) and (b)) and chest (c) computed tomography. A massive, floating thrombus (approximately 18 × 20 mm) extended from the right common iliac vein to the infrarenal inferior vena cava; an intra-arterial thrombus occluded a right lower lobe pulmonary artery.

A 40-year-old woman presented with a uterine cervical adenocarcinoma with metastasis to the liver, right kidney, pelvis, and sacral bone. She was referred to our department because an IVC thrombus was detected on contrast-enhanced abdominal CT. The patient's family history was unremarkable, but she did present with lower back pain and severe, bilateral leg edema. A physical examination showed a regular pulse rate of 91 beats/min, blood pressure of 132/90 mmHg, and a normal temperature of 35.9°C. Upon auscultation, the patient did not demonstrate any obvious murmur or rales. Her laboratory tests revealed severe anemia (hemoglobin level, 6.8 g/dL) and an increased D-dimer concentration (32.4 *μ*g/mL). Contrast-enhanced CT demonstrated a massive, floating thrombus, approximately 18 × 20 mm in size, extending from the right common iliac vein to the infrarenal IVC (Figures 2(a) and 2(b)); an intra-arterial thrombus occluding a right lower lobe pulmonary artery was also noted (Figure 2(c)). We considered the patient to have a high risk of recurrent PE, but administration of an effective and safe anticoagulant therapy was difficult due to her severe anemia. Thus, an IVC filter was implanted. Although PEs were successfully prevented, she succumbed to the uterine cervical adenocarcinoma approximately 1 month later.

### 2.3. Case 3

**Figure 3:**
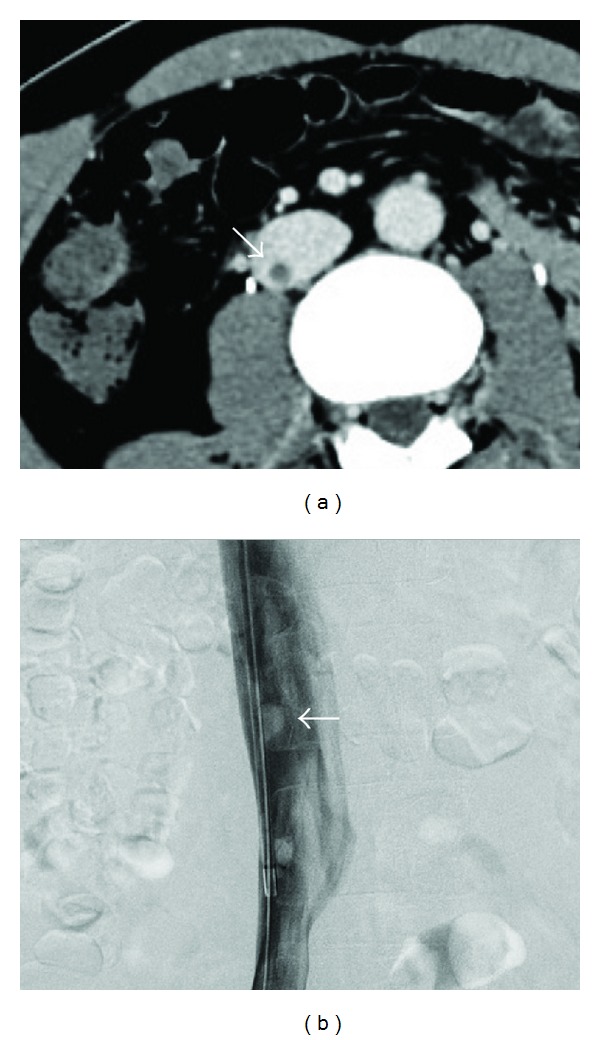
Contrast-enhanced computed tomography (a) and catheter-based venography (b). A small, polyp-like thrombus was observed at the infrarenal inferior vena cava.

A 37-year-old man with a liver abscess was referred to our department for management after the implantation of an IVC filter. The patient's family history was unremarkable, and the patient had not complained of chest pain, dyspnea, or lower back pain. His physical examination showed a regular pulse rate of 80 beats/min, blood pressure of 90/50 mmHg, and a normal temperature of 36.8°C. The patient did not demonstrate any obvious murmur or rales, but his laboratory tests revealed an increased D-dimer concentration (3.6 *μ*g/mL); the patient did not show any findings suggestive of congenital thrombotic disease. Contrast-enhanced CT and catheter-based venography demonstrated a small, polyp-like thrombus at the site of the infrarenal IVC (Figure 3), in absence of any IVC malformation. The patient was administered an anticoagulant therapy involving unfractionated heparin, and the IVC thrombus was completely resolved approximately 2 weeks later. He was discharged after removal of the filter.

## 3. Discussion

IVC thrombosis is a rare but significant complication in hospitalized patients. According to a report (1979–2005) from the United States, DVTs occur in 0.85% of hospitalized patients and 1.25% of these patients have IVC thrombosis. PEs occurred in 12% of the patients with IVC thrombosis, in the absence of DVTs at other sites [5]. However, relevant information regarding IVC thrombosis, especially about its morphology, remains scarce. In this report, our findings indicate that IVC thromboses can show at least three morphologies, that is, mural, floating, and small, polyp-like thromboses; this has not been reported previously. The clinical significance of the morphological characteristics of IVC thrombi could not be clarified based on the reported cases. However, we speculate that the floating and polyp-like thrombi may be more likely to cause PEs than the mural type because the risk of PE has been reported to be also higher in patients with free-floating thrombi in the lower-extremity deep veins [6]. Further studies in more patients are required to verify or refute this suggestion.

The classical physical signs of IVC thrombosis include bilateral leg swelling and dilated superficial abdominal wall collateral veins [7]; another sign preceding the development of these signs is lower back pain [8]. However, two of the present cases did not have significant clinical signs or symptoms, and the IVC thromboses were only incidentally diagnosed when the patients underwent contrast-enhanced CT. The etiology of DVTs includes long-haul flights, pregnancy, prolonged immobility, cancer, obesity, oral contraceptive use, thrombophilia, a history of VTE, and the presence of varicose veins [1]. IVC thromboses have also been reported to be frequently associated with cancer [4, 5]. Furthermore, extensive venous thromboses are frequently seen in postmortem liver abscesses [9], and IVC thromboses have been occasionally reported in patients with liver abscesses [10, 11]. Thus, physicians should keep in mind that such patients may have IVC thromboses as well as lower-extremity vein thromboses, even if they are asymptomatic.

In conclusion, IVC thromboses can manifest with at least three different morphologies, namely, mural, floating, and small, polyp-like thromboses. We believe that it is important to further elucidate the clinical significance of these findings in future studies in more patients.
